# Lipoprotein lipase links vitamin D, insulin resistance, and type 2 diabetes: a cross-sectional epidemiological study

**DOI:** 10.1186/1475-2840-12-17

**Published:** 2013-01-16

**Authors:** Yifan Huang, Xiaoxia Li, Maoqing Wang, Hua Ning, Lima A, Ying Li, Changhao Sun

**Affiliations:** 1 Department of Nutrition and Food Hygiene, Public Health College, Harbin Medical University, Harbin, P.R. China

**Keywords:** Lipoprotein lipase, Vitamin D, Diabetes, Insulin resistance, Lipid metabolism

## Abstract

**Background:**

Lipoprotein lipase (LPL) and serum 25-hydroxyvitamin D [25(OH)D] play important roles in the regulation of lipid metabolism. Although dyslipidemia is associated with insulin resistance (IR) and type 2 diabetes (T2D), there are limited data available regarding the relationship of LPL and 25(OH)D to IR and T2D at a population level. The objective of the present study is to investigate the associations of LPL and 25(OH)D with IR and T2D in a Chinese population.

**Methods:**

The study cohort consisted of 2708 subjects (1326 males, 1382 females; mean age 48.5 ± 12.6 years) in main communities of Harbin, China. Serum 25(OH)D, LPL, free fatty acids (FFAs), fasting glucose (FG), fasting insulin, lipid profile, apoA and apoB concentrations were measured.

**Results:**

Serum 25(OH)D concentration was positively associated with LPL (β = 0.168, P *<* 0.001). LPL was inversely associated with IR and T2D. Subjects in the lowest quartile of LPL had the highest risk of IR [odds ratio (OR) = 1.85, 95% CI = 1.22-2.68] and T2D (OR = 1.65, 95% CI = 1.14-2.38). Serum 25(OH)D was also inversely associated with IR and T2D. Vitamin D deficiency [25(OH)D < 20 ng/ml] was associated with an increasing risk of IR (OR = 1.91, 95% CI = 1.23-2.76) and T2D (OR = 2.06, 95% CI = 1.37-3.24). The associations of 25(OH)D with IR and T2D were attenuated by further adjustment for LPL.

**Conclusions:**

LPL is associated with serum 25(OH)D, IR and T2D in the Chinese population. These results suggest a potential mediating role of LPL in the associations of 25(OH)D with IR and T2D.

## Background

Lipoprotein lipase (LPL) is a member of the so-called lipase superfamily which includes hepatic lipase, pancreatic lipase and LPL itself [1]. Although it is mainly synthesized by the parenchymal cells in adipose, skeletal and cardiac muscle, LPL has its physiological site of action at the capillary endothelial cell surface where the enzyme catalyzes the lipolysis of triglyceride (TG) to provide free fatty acids (FFAs) and 2-monoacylglycerol for tissue utilization [2,3]. Therefore, LPL plays a central role in lipid metabolism and is widely distributed in various tissues. In addition to its effect on the lipid metabolism, LPL is also directly or indirectly implicated in some pathophysiological conditions such as insulin resistance (IR) and type 2 diabetes (T2D). Reduction of LPL is observed in patients with T2D and individuals with IR [4-6]. Low LPL activity accompanied by high TG was observed in diabetic dyslipidemia [7].

**Figure 1:**
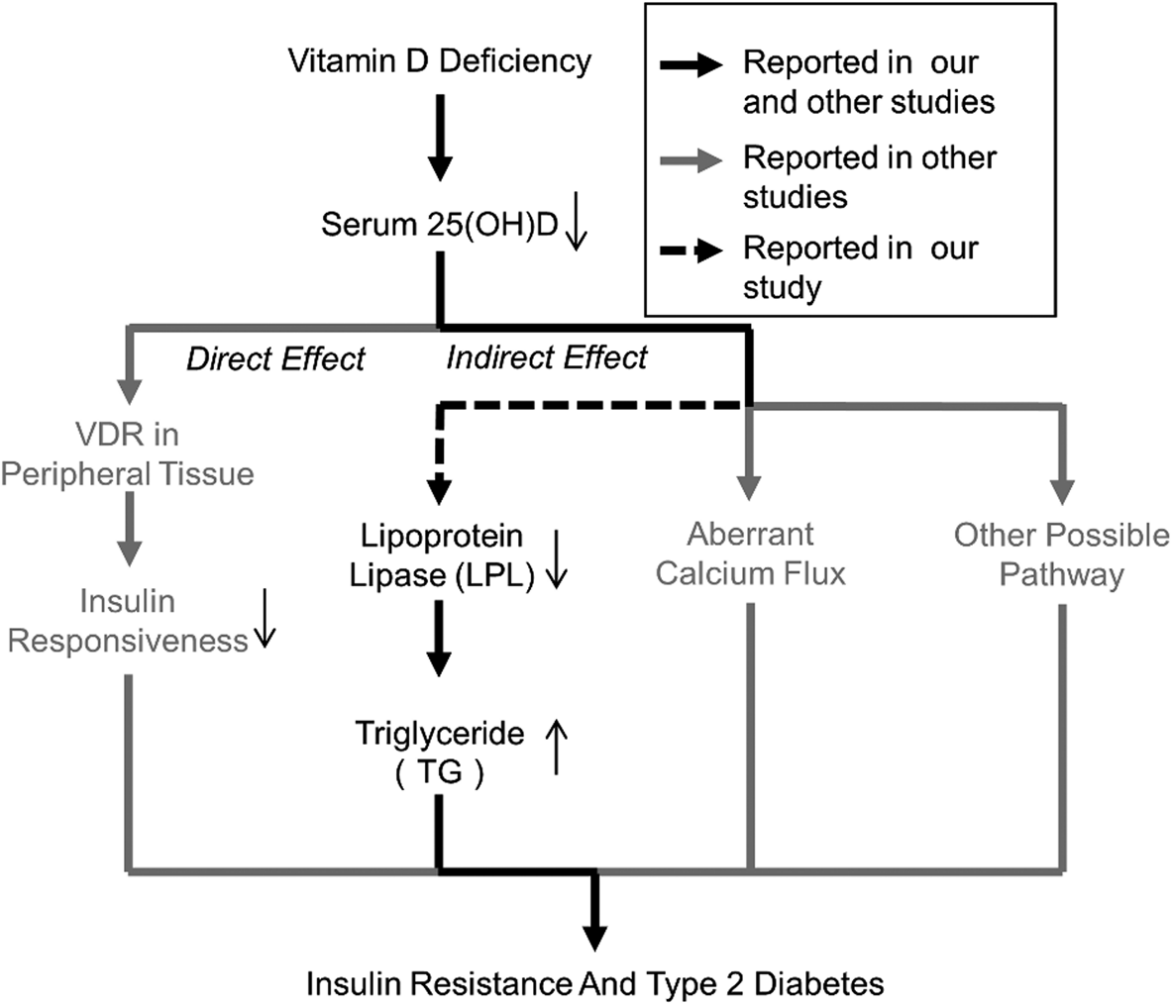
**Putative scheme of effect of vitamin D on insulin resistance and type 2 diabetes.** Vitamin D deficiency influences the pathogenesis of insulin resistance and type 2 diabetes by direct effect such as decreased binding to vitamin D receptor (VDR), and indirect effect such as aberrant calcium flux*.* Reductive serum 25(OH)D concentration was accompanied with decreased LPL and increased TG in our study, which might be associated with insulin resistance and type 2 diabetes.

In addition to LPL, vitamin D also plays an important role in the regulation of lipid metabolism. Over the past decades, numerous studies have shown that individuals with vitamin D deficiency are at an increasing risk for a disorder of lipid metabolism [8,9]; serum 25-hydroxyvitamin D [25(OH)D], the main circulating form of vitamin D, is significantly associated with lipid metabolism [10].

Despite a large body of literature in this regard, the relationship between LPL and vitamin D is not fully understood. Existing evidence suggested that treatment with 1,25-Dihydroxyvitamin D [1,25(OH)_2_D], the active form of vitamin D, could induce the LPL expression in cells [11]; however, few epidemiological studies have been so far designed to investigate the association between vitamin D and LPL at a population level. Furthermore, although it is known that dyslipidemia is associated with IR and T2D, information is limited on the association between LPL, 25(OH)D, IR and T2D in population studies. Importantly the role of LPL in linking 25(OH)D, IR and T2D is largely unknown. The objective of this cross-sectional epidemiologic study is to examine the relationship among LPL, 25(OH)D, IR and T2D in the northeastern Chinese population.

## Methods

### Study cohort

The source population consisted of 8127 adults, aged 20 to 74 years, who were recruited from main communities of Harbin, China [12]. In the current study, 3000 individuals (1500 males, 1500 females) were randomly selected from the source population between August and October 2008. After 292 subjects were excluded, the final study sample comprised a total of 2708 subjects (1326 males, 1382 females; mean age 48.5 ± 12.6 years). The exclusion criteria were any disorders or history known to alter vitamin D metabolism; drug or alcohol abuse or dependence; severe liver diseases including hepatitis, cirrhosis, or malignancy; under regular medication for controlling blood lipids; no signed informed consent. There were no significant differences between participants (2708 subjects) and the source population (8127 subjects) in age and BMI. Design and all procedures of the present study were in accordance with the Declaration of Helsinki. This study was approved by the Ethics Committee of the Harbin Medical University, and all subjects provided signed informed consent.

### Data collection

Demographic and lifestyle data were obtained using a general structured questionnaire. Body mass index (BMI, kg/m^2^) was calculated as weight in kilograms divided by the square of the height in meters. The physical activity level (PAL) was calculated with the formula from the American Institute of Medicine [13]. Blood samples were collected in tubes from each subject after fasting for 10 hours for laboratory analyses of study variables. The blood samples were immediately centrifuged at 2500 g for 15 min to obtain serum which was instantly cooled down, stored at −80 centigrade. Fasting serum glucose (FG) and 2-h post-load (75 g sugar) glucose (PG) for oral glucose tolerance test (OGTT) were measured quantitatively by the glucose oxidase method. Concentrations of total cholesterol (TC), high- (HDL) and low-density lipoprotein (LDL) cholesterols, TG, FFAs, apoA and apoB were assessed with standard enzymatic methods in an auto-analyzer (AUTOLAB PM 4000, AMS Corporation, Rome, Italy). Serum insulin was measured with an auto-analyzer using commercial kits (Centaur, Bayer Corporation, Bayer Leverkusen, Germany). LPL concentration in the post-heparin serum collected in 10 minutes after intravenous injection of 30 IU/kg heparin was measured by enzyme-linked immunosorbent assay (ELISA) using commercially available ELISA Kits (R&D System, USA). The inter-assay coefficient and intra-assay coefficient of LPL were 6.28% and 7.04%, respectively. Homeostasis model of assessment of insulin resistance (HOMA-IR) was calculated as HOMA-IR = Fasting glucose (mmol/L) × Fasting insulin (mU/L)/22.5. Serum 25(OH)D concentrations were determined by ACQUITY Ultra Performance Liquid Chromatography (Waters, USA). The inter-assay coefficient and intra-assay coefficient of 25(OH)D at 20 ng/mL were 4.69% and 3.72%, respectively. IR was defined as HOMA-IR higher than 2.50. T2D was defined as FG ≥ 7.0 mmol/L, or PG ≥ 11.1 mmol/L on a 2-h OGTT [14], or a diagnosis of diabetes. Vitamin D deficiency was defined as serum 25(OH)D concentrations < 20 ng/ml (50 nmol/L), insufficiency 21-29 ng/ml, and sufficiency ≥ 30 ng/ml (75 nmol/L) [15]. Suboptimal D status was defined as vitamin D deficiency or vitamin D insufficiency.

### Statistical analysis

Characteristics of study variables were presented as mean ± SD, or median for continuous variables and as percentage for categorical variables. Log-transformation was performed to improve normality of the distribution where necessary. Analysis of covariance (ANCOVA) was used to assess differences in study variables between subgroups classified by levels of LPL and 25(OH)D, with post hoc Bonferroni's correction for multiple comparisons. The associations between 25(OH)D and continuous variables such as LPL and FFAs were tested by multivariable linear regression analysis, adjusted for covariates (age, sex, BMI, PAL, smoking and alcohol use). The associations of serum 25(OH)D and LPL with IR and T2D were tested by logistic regression analysis, adjusted for the above covariates. In order to evaluate the influence of individual covariates (age, sex, BMI, PAL, smoking and alcohol use) on the association parameters (ORs), logistic regression models were performed separately by excluding the covariates one by one from the model and including the remaining variables. The changes in OR values reflect the influence of the variable removed from the model on the particular association. Statistical analyses were performed with SPSS software version 16.0 (SPSS Inc., Chicago, IL), and P value < 0.05 was considered statistically significant.

## Results

### Characteristics of study variables

The characteristics for study variables are shown in Table 1 by sex groups. Mean serum 25(OH)D concentration of the total sample was 25.4 ± 6.5 ng/ml. The prevalence of suboptimal vitamin D (vitamin D deficiency and insufficiency), IR and T2D in 2708 subjects was 74.7%, 19.5% and 15.9%, respectively.

**Table 1:** **Demographic and clinical characteristics for males and females**^**a**^

	**Males**	**Females**	***P***
N	1326	1382	
**Basic characteristics**			
Age, y	47.6 ± 11.3	49.4 ± 13.1	<0.001
BMI, kg/m^2^	25.7 ± 3.8	25.2 ± 3.3	0.002
Physical activity level	1.45 ± 0.40	1.49 ± 0.42	0.011
Insulin Resistance, n (%)	288 (21.7)	240 (17.4)	0.004
Type 2 diabetes, n (%)	243 (18.3)	189 (13.7)	<0.001
Obesity, n (%)	273 (20.6)	283 (20.5)	0.943
Alcohol use, n (%)	797 (60.1)	170 (12.3)	<0.001
Smoking, n (%)			<0.001
Never	541 (40.8)	1273 (92.1)	
Past	164 (12.4)	23 (1.7)	
Current	621 (46.8)	86 (6.2)	
**Clinical variables**			
25(OH)D, ng/ml	25.7 ± 6.7	25.1 ± 6.4	0.017
FG, mmol/L	5.1 (3.6-9.2)	4.9 (3.4-10.3)	0.054
PG, mmol/L	7.9 (3.8-14.5)	7.6 (3.5-13.6)	0.011
HbAlc, %	4.3 (3.4-11.7)	3.9 (3.3-11.5)	0.002
Insulin, mU/L	6.1 (1.5-17.5)	6.0 (1.4-16.8)	0.051
HOMA-IR	1.4 (0.3-6.2)	1.3 (0.3-6.0)	0.011
TC, mmol/L	4.8 ± 1.1	4.9 ± 1.2	0.024
TG, mmol/L	1.7 (0.5-6.1)	1.5 (0.4-5.9)	<0.001
HDL-C, mmol/L	1.5 (0.8-2.3)	1.5 (0.7-2.4)	0.286
LDL-C, mmol/L	3.0 (0.6-5.2)	2.9 (0.5-5.4)	0.118
apoA, mmol/L	1.8 ± 0.4	2.0 ± 0.4	<0.001
apoB, mmol/L	0.8 ± 0.2	0.7 ± 0.3	<0.001
FFAs, μmol/L	723.8 ± 293.7	729.4 ± 265.4	0.602
LPL, U/L	653.1 ± 187.8	664.3 ± 193.5	0.127

^**a**^ Data are presented as n(%), means ± SD or median (range). χ^2^ test or ANOVA were adopted to compare differences between males and females.

Abbreviations: FFAs, free fatty acids; FG, fasting serum glucose; HOMA-IR, Homeostasis model of assessment - insulin resistance; LPL, lipoprotein lipase; PG, 2 h post-load glucose; TC, total cholesterol; HDL-C, high-density lipoprotein cholesterol; LDL-C, low-density lipoprotein cholesterol; TG, triglyceride.

### Differences in variables among quartiles of LPL

Table 2 shows differences in study variables among LPL quartile groups. BMI values and prevalence of obesity decreased with increasing LPL quartiles, but the trend was borderline significant. All metabolic variables had significantly increasing or decreasing trends across LPL quartile groups except for insulin, TC and apoB. LDL-C had a marginally significant decreasing trend across LPL quartile groups (P = 0.068).

**Table 2:** **Characteristics of study variables by lipoprotein lipase quartile groups**^**a**^

	**Quartiles of LPL (U/L)**				
	**Q 1 (< 532.8)**	**Q 2 (532.9 - 653.2)**	**Q 3 (653.3 - 778.6)**	**Q 4 (> 778.6)**	***P***
**N**	677	677	677	677	
**Basic characteristics**					
Age, y	49.2 ± 12.6	47.9 ± 12.3	48.3 ± 10.1	48.6 ± 11.4	0.213
BMI, kg/m^2^	25.6 ± 3.8	25.2 ± 3.5	25.3 ± 3.6	25.1 ± 3.1	0.053
Physical activity level	1.49 ± 0.39	1.46 ± 0.47	1.50 ± 0.44	1.47 ± 0.38	0.283
Obesity, n (%)	158 (23.3)	142 (21.0)	135 (19.9)	121 (17.9)	0.093
**Metabolic variables**					
25(OH)D, ng/ml	23.1 ± 6.7	24.6 ± 6.3	26.4 ± 6.2	27.8 ± 7.1	<0.001
FG, mmol/L	5.2 (3.5-11.5)	5.0 (3.3-10.8)	4.9 (2.8-10.7)	4.9 (3.0-11.2)	0.021
PG, mmol/L	8.0 (3.6-13.3)	7.7 (3.5-12.7)	7.6 (3.1-12.1)	7.4 (3.0-12.4)	0.008
HbAlc, %	4.3 (3.2-11.9)	4.2 (3.3-12.5)	4.0 (3.2-11.4)	3.9 (3.2-11.7)	<0.001
Insulin, mU/L	6.0 (1.4-17.6)	5.8 (1.5-17.9)	5.9 (1.6-17.7)	5.8 (1.4-18.1)	0.149
HOMA-IR	1.5 (0.3-6.6)	1.4 (0.4-6.4)	1.4 (0.2-6.0)	1.3 (0.3-5.9)	<0.001
TC, mmol/L	4.8 ± 1.2	4.7 ± 1.1	4.9 ± 1.3	4.8 ± 1.2	0.201
TG, mmol/L	1.9 (0.6-6.2)	1.8 (0.5-6.0)	1.5 (0.7-5.6)	1.5 (0.6-5.9)	<0.001
HDL-C, mmol/L	1.4 (0.7-2.1)	1.4 (0.6-2.4)	1.5 (0.7-2.3)	1.5 (0.8-2.5)	0.032
LDL-C, mmol/L	3.0 (0.7-5.3)	3.0 (0.6-5.1)	2.9 (0.5-4.9)	2.9 (0.5-5.2)	0.068
apoA, mmol/L	1.9 ± 0.4	1.9 ± 0.5	2.0 ± 0.4	2.1 ± 0.5	<0.001
apoB, mmol/L	0.8 ± 0.2	0.8 ± 0.3	0.8 ± 0.2	0.8 ± 0.3	0.470
FFAs, μmol/L	776.4 ± 293.2	741.3 ± 295.4	713.2 ± 289.7	685.4 ± 278.5	<0.001

^**a**^ Data are presented as n(%), means ± SD or median (range). χ^2^ test or ANOVA were adopted to compare the difference among LPL quartile groups.

Abbreviations: FFAs, free fatty acids; FG, fasting serum glucose; LPL, lipoprotein lipase; PAL, Physical activity level; PG: 2 h post-load glucose; TC, total cholesterol; HDL-C, high-density lipoprotein cholesterol; LDL-C, low-density lipoprotein cholesterol; TG, triglyceride.

### The association of serum 25(OH)D with LPL and other metabolic variables

The effects of 25(OH)D and LPL on metabolic variables were further examined with ANCOVA as summarized in Table 3. After adjusted for age, sex, BMI, PAL, smoking and alcohol consumption, subjects with vitamin D deficiency indicated significant differences in metabolic variables compared with vitamin D sufficiency group except for insulin, TC, LDL-C and apoB. In subjects with vitamin D insufficiency, selected metabolic variables had significant differences when compared with vitamin D sufficiency group. Of interest, subjects in both vitamin D deficiency and insufficiency groups had significantly lower LPL values than those in vitamin D sufficiency group (P < 0.05).

**Table 3:** Means or medians by serum 25 (OH)D status and regression coefficients of serum 25(OH)D on metabolic variables

**Dependent variables**	**Serum 25 (OH)D status**^**a**^			**Regression model 1**^**b**^		**Regression model 2**^**c**^	
	**Deficiency (<20 ng/ml)**	**Insufficiency (20-29 ng/ml)**	**Sufficiency (>30 ng/ml)**	**β**	***P***	**β**	***P***
**Subjects (n)**	422	1602	684				
**FG, mmol/L**	5.3 (3.6-11.5)*	5.0 (3.1-9.3)	4.9 (2.7-8.8)	−0.086	0.011	−0.055	0.034
**PG, mmol/L**	8.5 (3.7-13.9)*	7.8 (3.5-12.4)	7.7 (3.2-12.1)	−0.091	0.002	−0.058	0.027
**HbAlc, %**	4.4 (3.3-11.5)**	4.2 (3.2-11.8)**	4.0 (3.3-11.6)	−0.120	<0.001	−0.082	0.012
**Insulin, mU/L**	5.9 (1.5-16.9)	6.1 (1.4-17.8)	6.0 (1.6-18.1)	−0.045	0.051	−0.042	0.058
**HOMA-IR**	1.5 (0.3-6.5)**	1.4 (0.3-6.1)*	1.3 (0.3-5.8)	−0.127	<0.001	−0.048	0.047
**TC, mmol/L**	5.0 ± 1.2	4.8 ± 1.3	4.8 ± 1.2	−0.014	0.532	−0.015	0.525
**TG, mmol/L**	1.8 (0.8-6.3)*	1.6 (0.5-6.1)	1.5 (0.6-5.8)	−0.093	0.001	−0.043	0.056
**HDL-C, mmol/L**	1.4 (0.7-2.2)*	1.5 (0.8-2.4)	1.5 (0.8-2.3)	0.117	<0.001	0.084	0.011
**LDL-C, mmol/L**	3.0 (0.8-5.3)	2.9 (0.7-5.2)	2.9 (0.5-5.5)	−0.021	0.431	−0.018	0.513
**apoA, mmol/L**	1.9 ± 0.4*	2.0 ± 0.4	2.0 ± 0.3	0.089	0.005	0.052	0.028
**apoB, mmol/L**	0.8 ± 0.2	0.8 ± 0.3	0.8 ± 0.2	0.024	0.353	0.022	0.358
**FFAs, μmol/L**	778.6 ± 284.8**	734.5 ± 306.4*	672.8 ± 297.5	−0.145	<0.001	−0.077	0.021
**LPL, U/L**	615.3 ± 192.4**	658.8 ± 196.6*	691.2 ± 194.1	0.168	<0.001	NA	NA

^**a**^ Means ± SD or medians ( range). * *P* < 0.05, ** *P* < 0.01, compared with vitamin D sufficiency group by ANCOVA, adjusted for age, sex, BMI, physical activity level, smoking and alcohol consumption.

^**b**^ Multivariable linear regression analyses model 1, with 25(OH)D concentration as the independent variable, adjusted for age, sex, BMI, physical activity level, smoking and alcohol consumption.

^**c**^ Multivariable linear regression analyses model 2, with 25(OH)D concentration as the independent variable adjusted for covariates in model 1 + LPL.

Abbreviations: FFAs, free fatty acids; FG, fasting serum glucose; LPL, lipoprotein lipase; PAL, Physical activity level; PG: 2 h post-load glucose; TC, total cholesterol; HDL-C, high-density lipoprotein cholesterol; LDL-C, low-density lipoprotein cholesterol; TG, triglyceride; β, regression coefficients.

In multivariable linear regression analysis model 1, with 25(OH)D as the independent variable, adjusted for the same confounding factors in ANCOVA above, 25(OH)D was significantly associated with all metabolic variables except for TC, LDL-C and apoB, and marginally associated with insulin (P = 0.051). In regression model 2, inclusion of LPL did not substantially change the regression parameters; however, the association between TG and 25(OH)D was weakened when LPL was included in model 2.

### The relationship of vitamin D and LPL to IR and T2D

Table 4 presents association parameters (OR and 95% CI) of serum 25(OH)D and LPL with IR and T2D from logistic regression models. In model 1, subjects with vitamin D deficiency were 1.91 and 2.06 times more likely to develop IR and T2D, respectively. In model 2 these association parameters were attenuated by the inclusion of LPL which was positively associated with serum 25(OH)D. Lower LPL was significantly associated with a higher risk of IR and T2D. The associations became stronger with decreasing quartiles of LPL. Compared with subjects in the top LPL quartile group, the risk was 1.85 times higher for IR and 1.65 times higher for T2D among those in the bottom LPL quartile group.

**Table 4:** Associations of serum 25(OH)D and LPL with IR and T2D

	**25(OH)D (ng/ml)**				**Quartiles of LPL (U/L)**					
	**Deficiency**	**Insufficiency**	**Sufficiency**	**Q 1**	**Q 2**	**Q 3**	**Q 4**
	**(< 20.0)**	**(20.1-29.9)**	**(≥ 30.0)**	**(≤ 532.8)**	**(532.9-653.2)**	**(653.3-778.6)**	**(> 778.6)**
**IR, OR (95% CI)**							
Cases/Non-cases	101/321	324/1278	103/581	159/518	143/534	128/549	98/579
Model 1	1.91 (1.23-2.76)	1.42 (1.04-1.87)	1.00 (reference)	1.85 (1.22-2.68)	1.51 (1.03-2.25)	1.32 (0.91-1.93)	1.00 (reference)
Model 2	1.35 (1.12-2.45)	1.13 (1.02-1.82)	1.00 (reference)	NA	NA	NA	NA
**T2D, OR (95% CI)**							
Cases/Non-cases	87/335	269/1333	76/608	124/553	113/564	101/576	94/583
Model 1	2.06 (1.37-3.24)	1.57 (1.12-2.29)	1.00 (reference)	1.65 (1.14-2.38)	1.41 (1.07-2.25)	1.29 (0.84-1.96)	1.00 (reference)
Model 2	1.42 (1.09-2.68)	1.10 (1.03-1.93)	1.00 (reference)	NA	NA	NA	NA

Model 1, adjusted for age, sex, BMI, PAL, smoking and alcohol consumption.

Model 2, adjusted for covariates in model 1 + LPL.

25(OH)D, 25-hydroxyvitamin D; IR, insulin resistance; T2D, type 2 diabetes; LPL, lipoprotein lipase; PAL, physical activity level.

### The effects of demographic and lifestyle variables on association parameters

In the evaluation analysis of the influence of individual covariates (age, sex, BMI, PAL, smoking and alcohol use) on the association parameters (ORs) in Table 4, BMI showed a marked influence on the association of vitamin D with IR and T2D. Other covariates did not substantially affect the OR values.

## Discussion

Among 2708 northeastern Chinese adults in the current study, the main findings were that LPL was positively associated with serum 25(OH)D concentration; LPL and serum 25(OH)D concentration were inversely associated with the prevalence of IR and T2D; whereas the associations of 25(OH)D with IR and T2D were attenuated by further adjusted for LPL. To the best of our knowledge, this is the first epidemiological study reporting the association between serum 25(OH)D and LPL in the Chinese population.

### Vitamin D and type 2 diabetes

The rapid economic development has been accompanied by westernization of lifestyle behaviors and an increasing epidemic of obesity and metabolic syndrome in China during the past couple of decades. Consequently, the incidence and prevalence of T2D has been rapidly rising and has become a major public health problem in China as well as in developed countries [16-18]. Previous survey indicated the prevalence of T2D is 8.8% in the source population (n=8940) recruited in Harbin, China [19]. In the present study cohort, the percentage of T2D is 15.9%. Different source population size and the exclusion of 292 non-diabetic subjects from the present study accounted for the difference in the two prevalence rates. Furthermore, the sampling bias may partially explain the difference.

Individuals with low serum 25(OH)D concentration are at increasing risk for dyslipidemia, IR and T2D [20-22]. However, the relationship between vitamin D status and metabolic syndrome needs to be further investigated since the results from previous studies are controversial. For instance, studies of the British Cohort and nationally representative sample of the U.S. population both reported an inverse association between serum 25(OH)D concentration and metabolic syndrome [21,23], but another study in obese population did not find such a association [24]. Considering a strong link between diabetes and metabolic syndrome, the inverse association between 25(OH)D and T2D observed in our study supported the notion that vitamin D is associated with metabolic syndrome.

The favorable effect of improvement in vitamin D on T2D could be explained by its direct effect on insulin action. 1,25(OH)_2_D enhances insulin responsiveness for glucose transport by stimulating the expression of insulin receptor in peripheral tissue [25]. Besides, supplementation of vitamin D in experimental diabetic models significantly improved the concentration and integrity of the elastic lamellae in the medial layer of the aorta, and prevented fragmentation of elastic fibers in the aortic media [26].

Recently, a prospective intervention study indicated that 18 months vitamin D supplementation on adult patients with T2D significantly improved lipid profile with a favorable change in HDL/LDL ratio [27]. Considering that significant associations between serum 25(OH)D and lipid profile such as TG and HDL were also observed in our study, it is possible that the disorder of lipid metabolism may partially mediate the pathogenesis of IR and T2D caused by vitamin D deficiency. Therefore, LPL was introduced and investigated due to its primary role in the overall lipid metabolism.

### LPL linking vitamin D and type 2 diabetes

As a major enzyme responsible for lipolysis of circulating lipoproteins, LPL can be activated by PPAR through agonists, and inactivated by angiopoietin-like protein 4 [28]. Research carried out in the population over the past few years suggested an inverse association between LPL and TG [29]. Despite the fact that capillary endothelial cell surface is the physiological site of LPL-mediated hydrolysis reaction, adipose tissue is one of the most dominant sites for the synthesis of LPL [2,30]. Previous *in vitro* studies have shown that incubation with 1,25(OH)_2_D significantly increased LPL activity and mRNA in cultured adipocytes [31] and induced LPL expression in 3 T3-L1 cells [11]. If *in vivo* studies showed a similar effect of 1,25(OH)_2_D on LPL expression, it would be helpful to investigate the role of LPL in the association between vitamin D and glucose-lipid metabolism. Serum 25(OH)D concentration was positively associated with LPL in the present study. This result is helpful to explain the inverse association between serum 25(OH)D and TG, since hydrolysis reaction mediated by LPL will result in a decrease of TG in serum (Figure 1). Although supported by previous *in vitro* studies, the observed association between 25(OH)D and LPL in this population still needs to be confirmed in other studies.

Additionally, LPL was found to be inversely and independently associated with IR and T2D in the present study. Since further adjusted for LPL markedly attenuated the associations of 25(OH)D with IR and T2D, the finding suggested that associations of 25(OH)D with IR and T2D might be partially mediated by LPL. Certainly, further studies are needed to clarify this concept.

Serum FFAs concentration was also introduced in the present study. It is well established that abnormality of serum FFAs concentration plays an important role in the pathological incidence and development of IR and T2D, but data on the association between serum 25(OH)D and FFAs at a population level are still limited. In our study, serum 25(OH)D was inversely associated with FFAs even after adjusted for confounding factors. Despite the unknown mechanisms, previous *in vitro* studies provided the following two aspects to explain the association between 25(OH)D and FFAs. Firstly, activation of PPAR-δ by 1,25(OH)_2_D [32] will induce decreased FFAs concentrations because of increased fat oxidation and utilization of fatty acids by skeletal muscle [33]; secondly, treatment with 1,25(OH)_2_D could result in promotion of fatty acid synthesis and inhibition of lipolysis [34,35] in adipocytes, and hence the serum FFAs concentration was increased under vitamin D deficiency status.

### Prevalence of suboptimal vitamin D

High prevalence of suboptimal vitamin D has been a worldwide health problem. It is estimated that at least 30% of the general population in North America, Europe, Asia and Australasia has vitamin D deficiency [36]. However, Data from epidemiologic studies with large samples in northeastern China are still limited in this regard. Due to the high latitude of northeastern China, sunshine in this area is relatively inadequate, thus residents living in this area are at high risk of vitamin D deficiency [37]. According to the findings from previous survey [37], a majority of northeastern Chinese adults (>70%) was estimated to be in suboptimal vitamin D status. It is well known that the season when blood is collected is one of the influencing factors of vitamin D concentration. In the current study, all serum samples were collected between August and October when the sunshine is relatively abundant in this area. A comparison study on vitamin D concentrations in different seasons may provide more information in the northeastern area in China.

### Limitations and strengths

The present study is cross-sectional in nature; therefore the causal relationships between serum 25(OH)D, LPL, IR and T2D cannot be examined. Furthermore, LPL activity was not measured. The main strength of the present study is that the study cohort is representative of the source population with a large sample size. This is the first epidemiological study investigating the associations of serum LPL with 25(OH)D, IR and T2D in the population in China.

## Conclusions

The present study indicates that LPL is significantly associated with serum 25(OH)D, IR and T2D, adjusted for confounding factors. The associations of 25(OH)D with IR and T2D may be partially mediated by changes in LPL concentrations. Further *in vivo, vitro* and population studies are needed to replicate the findings from the current study.

## Abbreviations

ANOVA: analysis of variance; ANCOVA: analysis of covariance; BMI: body mass index; FFAs: free fatty acids; FG: fasting serum glucose; HOMA-IR: homeostatic model assessment of insulin resistance; IR: insulin resistance; LPL: lipoprotein lipase; OGTT: oral glucose tolerance test; PAL: Physical activity level; PG: 2-h post-load glucose; TC: total cholesterol; HDL: high-density lipoprotein cholesterol; LDL: low-density lipoprotein cholesterol; TG: triglyceride; T2D: type 2 diabetes; 25(OH)D: 25-hydroxyvitamin D.

## Competing interests

The authors declare that they have no competing interests.

## Authors’ contributions

CHS and YL designed the research; YFH, MQW, HN and LMA conducted research; YFH, XXL, YL and MQW analyzed and interpreted the data; YFH wrote the paper; YFH, XXL, YL and CHS had primary responsibility for final content. All authors read and approved the final manuscript.

## References

[B1] Hide WA Chan L Li WH Structure and evolution of the lipase superfamily Journal of lipid research 1992 33 2 167 178 1569370

[B2] Mead JR Irvine SA Ramji DP Lipoprotein lipase: structure, function, regulation, and role in disease J Mol Med (Berl) 2002 80 12 753 769 12483461 10.1007/s00109-002-0384-9

[B3] Goldberg IJ Merkel M Lipoprotein lipase: physiology, biochemistry, and molecular biology Frontiers in bioscience: a journal and virtual library 2001 6 D388 D405 11229871 10.2741/goldberg

[B4] Hanyu O Miida T Kosuge K Ito T Soda S Hirayama S Wardaningsih E Fueki Y Obayashi K Aizawa Y Preheparin lipoprotein lipase mass is a practical marker of insulin resistance in ambulatory type 2 diabetic patients treated with oral hypoglycemic agents Clinica chimica acta; international journal of clinical chemistry 2007 384 1-2 118 123 10.1016/j.cca.2007.06.015 17651713

[B5] Eckel RH Yost TJ Jensen DR Alterations in lipoprotein lipase in insulin resistance International journal of obesity and related metabolic disorders: journal of the International Association for the Study of Obesity 1995 19 Suppl 1 S16 S21 7550532

[B6] Eriksson JW Buren J Svensson M Olivecrona T Olivecrona G Postprandial regulation of blood lipids and adipose tissue lipoprotein lipase in type 2 diabetes patients and healthy control subjects Atherosclerosis 2003 166 2 359 367 12535750 10.1016/s0021-9150(02)00366-0

[B7] Taskinen MR Lipoprotein lipase in diabetes Diabetes/metabolism reviews 1987 3 2 551 570 3552532 10.1002/dmr.5610030208

[B8] Botella-Carretero JI Alvarez-Blasco F Villafruela JJ Balsa JA Vazquez C Escobar-Morreale HF Vitamin D deficiency is associated with the metabolic syndrome in morbid obesity Clin Nutr 2007 26 5 573 580 17624643 10.1016/j.clnu.2007.05.009

[B9] Lu L Yu Z Pan A Hu FB Franco OH Li H Li X Yang X Chen Y Lin X Plasma 25-hydroxyvitamin D concentration and metabolic syndrome among middle-aged and elderly Chinese individuals Diabetes care 2009 32 7 1278 1283 19366976 10.2337/dc09-0209 PMC2699709

[B10] Delvin EE Lambert M Levy E O'Loughlin J Mark S Gray-Donald K Paradis G Vitamin D status is modestly associated with glycemia and indicators of lipid metabolism in French-Canadian children and adolescents The Journal of nutrition 2010 140 5 987 991 20237070 10.3945/jn.109.112250

[B11] Vu D Ong JM Clemens TL Kern PA 1,25-dihydroxyvitamin D induces lipoprotein lipase expression in 3 T3-L1 cells in association with adipocyte differentiation Endocrinology 1996 137 5 1540 1544 8612483 10.1210/endo.137.5.8612483

[B12] Huang L Xue J He Y Wang J Sun C Feng R Teng J He Y Li Y Dietary calcium but not elemental calcium from supplements is associated with body composition and obesity in Chinese women PloS one 2011 6 12 e27703 22163269 10.1371/journal.pone.0027703 PMC3233543

[B13] Trumbo P Schlicker S Yates AA Poos M Food, Nutrition Board of the Institute of Medicine TNA: Dietary reference intakes for energy, carbohydrate, fiber, fat, fatty acids, cholesterol, protein and amino acids Journal of the American Dietetic Association 2002 102 11 1621 1630 12449285 10.1016/s0002-8223(02)90346-9

[B14] World Health Organization International Diabetes Federation 2006 World Health Organization: Definition and diagnosis of diabetes mellitus and intermediate hyperglycaemia: report of a WHO/IDF consultation. Geneva

[B15] Holick MF Siris ES Binkley N Beard MK Khan A Katzer JT Petruschke RA Chen E de Papp AE Prevalence of Vitamin D inadequacy among postmenopausal North American women receiving osteoporosis therapy The Journal of clinical endocrinology and metabolism 2005 90 6 3215 3224 15797954 10.1210/jc.2004-2364

[B16] Ujcic-Voortman JK Schram MT der Bruggen MA J-v Verhoeff AP Baan CA Diabetes prevalence and risk factors among ethnic minorities European journal of public health 2009 19 5 511 515 19587231 10.1093/eurpub/ckp096

[B17] Hanefeld M Koehler C Gallo S Benke I Ott P Impact of the individual components of the metabolic syndrome and their different combinations on the prevalence of atherosclerotic vascular disease in type 2 diabetes: the Diabetes in Germany (DIG) study Cardiovascular diabetology 2007 6 13 17462080 10.1186/1475-2840-6-13 PMC1871572

[B18] Liu S Wang W Zhang J He Y Yao C Zeng Z Piao J Howard BV Fabsitz RR Best L Prevalence of diabetes and impaired fasting glucose in Chinese adults, China National Nutrition and Health Survey, 2002 Preventing chronic disease 2011 8 1 A13 21159225 PMC3044024

[B19] Feng RN Zhao C Wang C Niu YC Li K Guo FC Li ST Sun CH Li Y BMI is strongly associated with hypertension, and waist circumference is strongly associated with type 2 diabetes and dyslipidemia, in northern Chinese adults Journal of epidemiology/ Japan Epidemiological Association 2012 22 4 317 323 22672914 10.2188/jea.JE20110120 PMC3798650

[B20] Holick MF Vitamin D deficiency N Engl J Med 2007 357 3 266 281 17634462 10.1056/NEJMra070553

[B21] Hypponen E Boucher BJ Berry DJ Power C 25-Hydroxyvitamin D, IGF-1, and Metabolic Syndrome at 45 Years of Age: A Cross-Sectional Study in the 1958 British Birth Cohort Diabetes 2007 57 2 298 305 18003755 10.2337/db07-1122

[B22] Ford ES Zhao G Li C Pearson WS Serum concentrations of vitamin D and parathyroid hormone and prevalent metabolic syndrome among adults in the United States Journal of diabetes 2009 1 4 296 303 20923530 10.1111/j.1753-0407.2009.00046.x

[B23] Ford ES Ajani UA McGuire LC Liu S Concentrations of serum vitamin D and the metabolic syndrome among U.S. adults Diabetes care 2005 28 5 1228 1230 15855599 10.2337/diacare.28.5.1228

[B24] Hjelmesaeth J Hofso D Aasheim ET Jenssen T Moan J Hager H Roislien J Bollerslev J Parathyroid hormone, but not vitamin D, is associated with the metabolic syndrome in morbidly obese women and men: a cross-sectional study Cardiovascular diabetology 2009 8 7 19187564 10.1186/1475-2840-8-7 PMC2644287

[B25] Pittas AG Lau J Hu FB Dawson-Hughes B The role of vitamin D and calcium in type 2 diabetes. A systematic review and meta-analysis. The Journal of clinical endocrinology and metabolism 2007 92 6 2017 2029 17389701 10.1210/jc.2007-0298 PMC2085234

[B26] Salum E Kampus P Zilmer M Eha J Butlin M Avolio AP Podramagi T Arend A Aunapuu M Kals J Effect of vitamin D on aortic remodeling in streptozotocin-induced diabetes Cardiovascular diabetology 2012 11 58 22631050 10.1186/1475-2840-11-58 PMC3398857

[B27] Al-Daghri NM Alkharfy KM Al-Othman A El-Kholie E Moharram O Alokail MS Al-Saleh Y Sabico S Kumar S Chrousos GP Vitamin D supplementation as an adjuvant therapy for patients with T2DM: an 18-month prospective interventional study Cardiovascular diabetology 2012 11 1 85 22809461 10.1186/1475-2840-11-85 PMC3461474

[B28] Makoveichuk E Sukonina V Kroupa O Thulin P Ehrenborg E Olivecrona T Olivecrona G Inactivation of lipoprotein lipase occurs on the surface of THP-1 macrophages where oligomers of angiopoietin-like protein 4 are formed Biochem Biophys Res Commun 2012 425 2 138 143 22820186 10.1016/j.bbrc.2012.07.048

[B29] Mingrone G Henriksen FL Greco AV Krogh LN Capristo E Gastaldelli A Castagneto M Ferrannini E Gasbarrini G Beck-Nielsen H Triglyceride-induced diabetes associated with familial lipoprotein lipase deficiency Diabetes 1999 48 6 1258 1263 10342813 10.2337/diabetes.48.6.1258

[B30] Braun JE Severson DL Regulation of the synthesis, processing and translocation of lipoprotein lipase The Biochemical journal 1992 287 Pt 2 337 347 1445192 10.1042/bj2870337 PMC1133170

[B31] Querfeld U Hoffmann MM Klaus G Eifinger F Ackerschott M Michalk D Kern PA Antagonistic effects of vitamin D and parathyroid hormone on lipoprotein lipase in cultured adipocytes J Am Soc Nephrol 1999 10 10 2158 2164 10505692 10.1681/ASN.V10102158

[B32] Mathieu C Gysemans C Giulietti A Bouillon R Vitamin D and diabetes Diabetologia 2005 48 7 1247 1257 15971062 10.1007/s00125-005-1802-7

[B33] Riserus U Sprecher D Johnson T Olson E Hirschberg S Liu A Fang Z Hegde P Richards D Sarov-Blat L Activation of peroxisome proliferator-activated receptor (PPAR)delta promotes reversal of multiple metabolic abnormalities, reduces oxidative stress, and increases fatty acid oxidation in moderately obese men Diabetes 2008 57 2 332 339 18024853 10.2337/db07-1318

[B34] Zemel MB Regulation of adiposity and obesity risk by dietary calcium: mechanisms and implications J Am Coll Nutr 2002 21 2 146S 151S 11999543 10.1080/07315724.2002.10719212

[B35] He YH Song Y Liao XL Wang L Li G Alima LG Li Y Sun CH The calcium-sensing receptor affects fat accumulation via effects on antilipolytic pathways in adipose tissue of rats fed low-calcium diets The Journal of nutrition 2011 141 11 1938 1946 21940515 10.3945/jn.111.141762

[B36] Holick MF High prevalence of vitamin D inadequacy and implications for health Mayo Clin Proc 2006 81 3 353 373 16529140 10.4065/81.3.353

[B37] Yan L Prentice A Zhang H Wang X Stirling DM Golden MM Vitamin D status and parathyroid hormone concentrations in Chinese women and men from north-east of the People's Republic of China European journal of clinical nutrition 2000 54 1 68 72 10694775 10.1038/sj.ejcn.1600895

